# Physiological and psychological outcomes of a community-based Kurort Health Walking program: A before–after study

**DOI:** 10.1371/journal.pone.0357733

**Published:** 2026-09-03

**Authors:** Saori Miura, Shinji Horikawa, Kana Morizono, Miyu Miyazaki, Hiroki Torii, Hiromi Kuroda, Emi Matsuura

**Affiliations:** 1 Graduate School of Biomedical Sciences, Nagasaki University, Sakamoto, Nagasaki, Japan; 2 Faculty of Nursing, St. Mary’s College, Kurume, Fukuoka, Japan; 3 Faculty of Health Sciences, Junshin Gakuen University, Minami-ku, Fukuoka, Japan; Japanese Academy of Health and Practice, JAPAN

## Abstract

**Objective:**

This study aimed to evaluate short-term physiological and psychological changes associated with participation in a community-based Kurort Health Walking program conducted in a forest environment.

**Patients and methods:**

This study was conducted as part of a public health program in Saikai City, Nagasaki, Japan. A total of 90 participants who attended certified Kurort Health Walking courses between March 2024 and March 2025 were included. Physiological parameters, including systolic and diastolic blood pressure and pulse rate, and psychological status were assessed before and after the walking session. Autonomic nervous activity was evaluated using heart rate variability indices, including the low-frequency/high-frequency ratio and high-frequency power, measured at four time points before, during, and after the walk. Mood states were assessed using validated questionnaires.

**Results:**

Systolic and diastolic blood pressure decreased significantly after the walking session (p < 0.001). Neither the low-frequency/high-frequency ratio nor high-frequency power showed significant changes across the measurement points. Negative mood states decreased significantly, whereas positive mood states, including vigor, friendliness, pleasure, and relaxation, increased, and anxiety decreased after walking (p < 0.001).

**Conclusion:**

Participation in the community-based Kurort Health Walking program was associated with short-term changes in physiological and psychological measures. However, because this study did not include a control group, the independent contributions of the forest environment, physical activity, and other contextual factors could not be distinguished.

## Introduction

Currently, more than half of the global population resides in urban areas, and this trend is projected to continue [1]. Urbanization has exposed individuals to chronic overstimulation and hectic lifestyles, raising concerns about its impact on mental health and cardiovascular disease [2,3]. Simultaneously, in rural areas, decreased physical activity due to automobile dependence has been linked to obesity and an increased risk of lifestyle-related diseases [4]. In this context, contact with nature is recognized as an essential means of stress recovery and health maintenance [5,6]. Thus, supporting health maintenance and promotion through natural environments is essential across populations in contemporary societies.

Activities in forest environments have been shown to improve cardiovascular function, such as lowering blood pressure and stabilizing heart rate, as well as to reduce stress, depressive symptoms, and anxiety [7,8]. In Germany, naturopathy, including forest therapy, is integrated into the public medical system and is implemented within an internationally advanced framework [9,10]. In Japan, “Kurort Health Walking,” based on the German Kurort system but adapted to the Japanese climate, culture, and national character, has gained significant attention [11].

Kurort is derived from the German words *kur* (cure, recuperation, or stay for health) and *ort* (place or region). Kurort Health Walking differs from typical walking in its emphasis on walking on sloped or sandy terrain, maintaining a sensation of slight coolness (cold air), avoiding excessive increases in heart rate, and experiencing natural elements such as wind and light [12,13].

Previous studies have suggested that Kurort Health Walking significantly reduces systolic blood pressure (SBP) and diastolic blood pressure (DBP) [14,15] and improves emotional states by reducing anger and depression while increasing vigor, pleasure, and relaxation [14–17]. Furthermore, while the highest efficacy is observed on sunny days, certain benefits, such as improved relaxation and reduced anxiety, are maintained even in rainy conditions [18]. Although previous studies have reported improvements in blood pressure and psychological outcomes following Kurort Health Walking, little evidence is available regarding autonomic nervous system responses, particularly heart rate variability indices such as the low-frequency/high-frequency (LF/HF) ratio and high-frequency (HF) power in real-world community settings. Evaluating autonomic nervous activity during community-based Kurort Health Walking programs may provide additional objective evidence regarding physiological responses associated with participation in natural environments.

Therefore, this study aimed to investigate short-term physiological and psychological changes associated with participation in a community-based Kurort Health Walking program conducted in forest environment. Specifically, physiological indices, including blood pressure, pulse rate, and autonomic nervous system activity, as well as psychological outcomes, were evaluated before and after the walking session. Evaluating these physiological and psychological changes may contribute to a better understanding of the short-term responses to community-based walking programs conducted in forest environments.

## Materials and methods

### Study design and participants

This study was conducted as a single-group observational pre–post study within an existing public health program implemented by the local government. Participants were recruited through the Saikai City Public Relations Magazine, the official municipal website, and the Nagasaki Prefecture walking application. Adults who voluntarily participated in one of two certified Kurort Health Walking courses organized by Saikai City, Nagasaki Prefecture, between March 20, 2024, and March 30, 2025, were included in this study. Individuals who were unable to complete the walking course or who declined to participate were excluded from the study. No restrictions were placed on previous participation history, medication use, or underlying diseases. Participation was limited to one session per course for each participant. Prior to the start of the study, the objectives and procedures were explained to all participants both orally and in writing, and written informed consent was obtained.

### Study fields

This study was conducted on two courses certified as “Kurort paths” by the Japan Kurort Research Institute in Saikai City.

#### Shihondo Park course.

**Fig 1:**
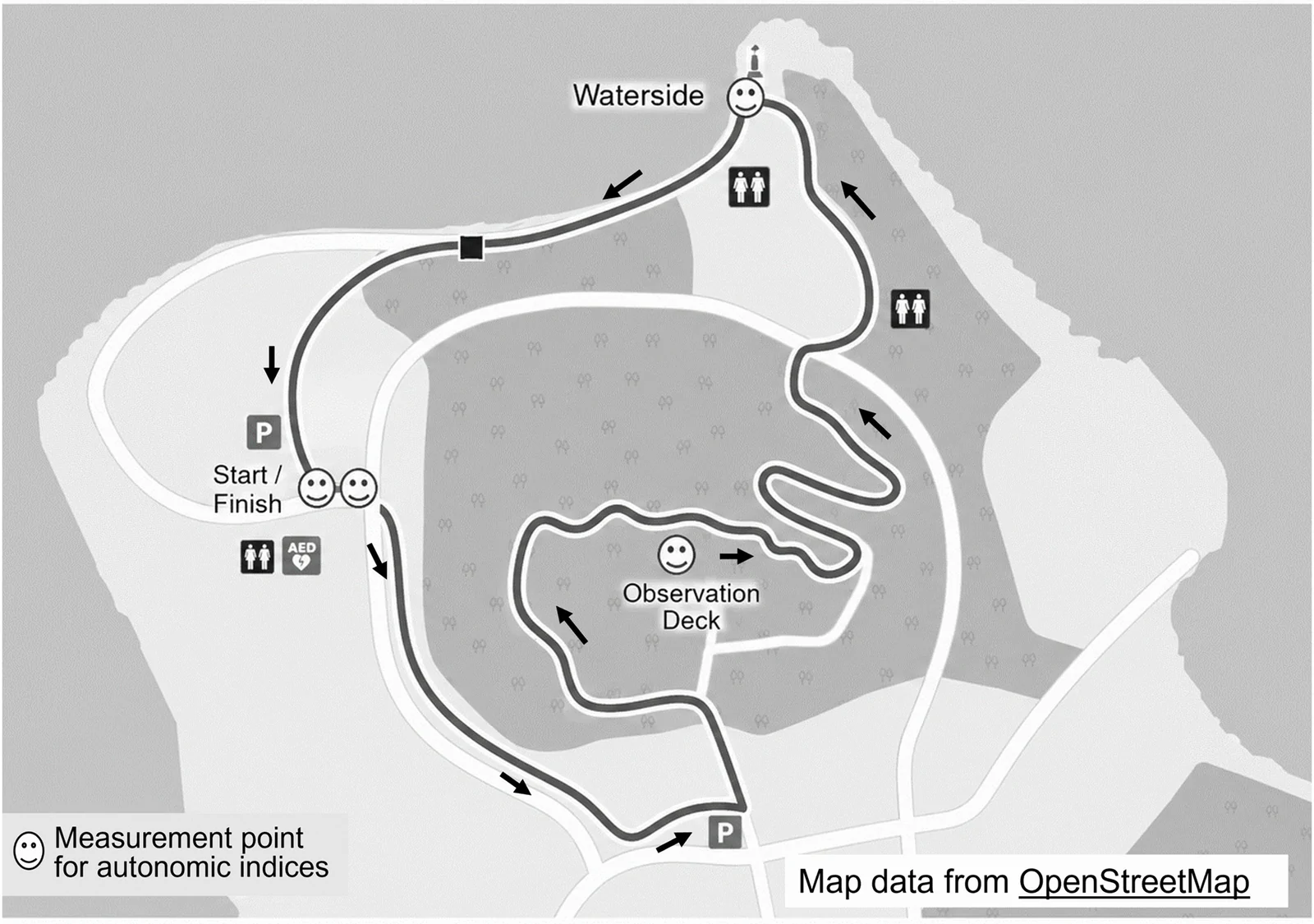
Map of the Shihondo Park course. The course has a total length of 1.83 km and a cumulative altitude gain of 74 m. Situated at the tip of a peninsula and surrounded by the sea on three sides, the course offers an expansive view. Base map and data from OpenStreetMap and OpenStreetMap Foundation. The walking route was added by the authors.

The Shihondo Park course is 1.83 km long with a cumulative altitude gain of 74 m. It is characterized by its location at the tip of the peninsula, surrounded by the sea on three sides, offering an expansive view (Fig 1).

#### Isanoura Park course.

**Fig 2:**
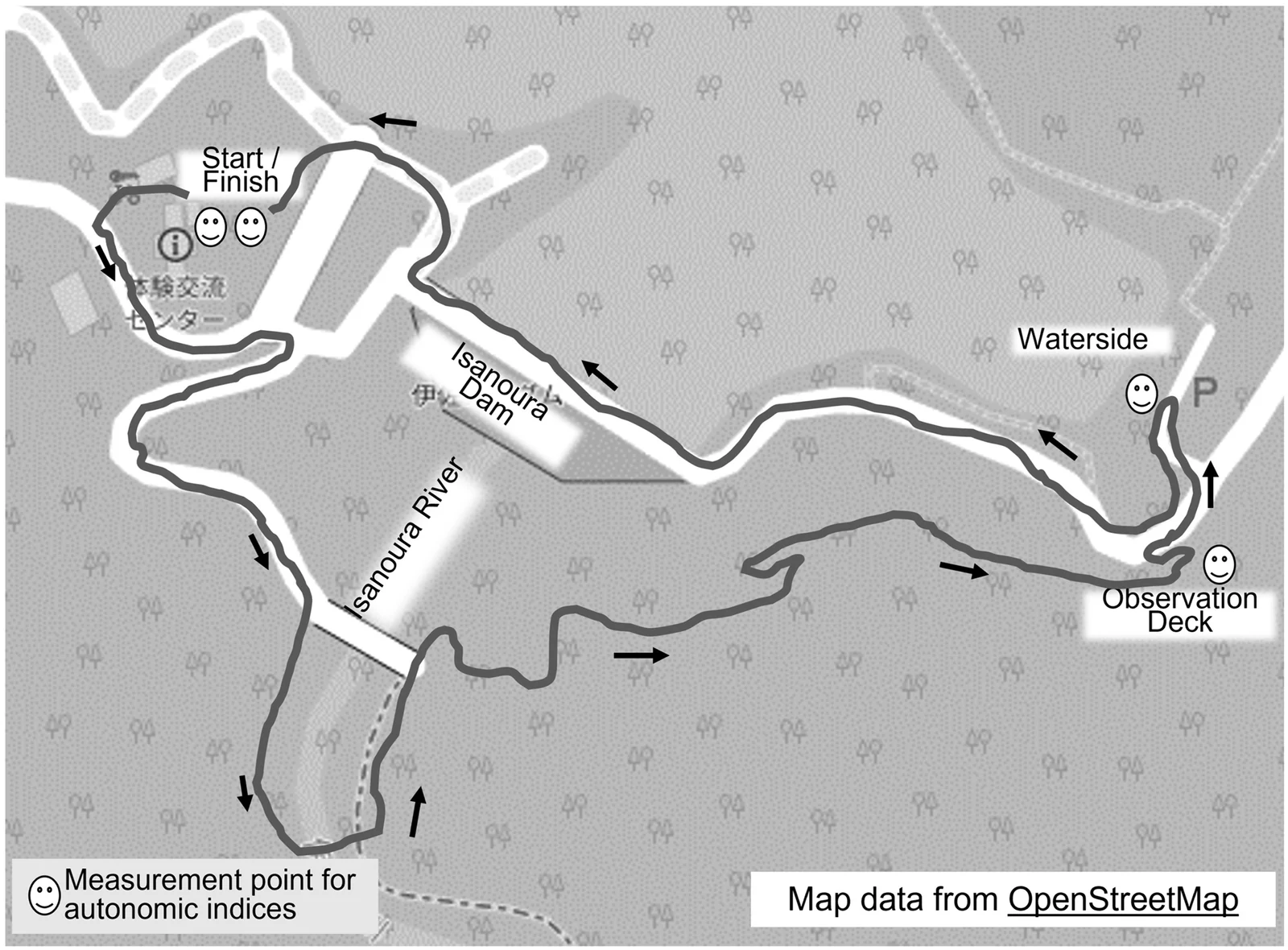
Map of the Isanoura Park course. The course has a total length of 2.1 km and a cumulative altitude gain of 73 m. The route passes through lush, natural forests surrounding the dam lake upstream of the Isanoura River, offering attractive scenery that changes with the seasons. Base map and data from OpenStreetMap and OpenStreetMap Foundation. The walking route was added by the authors.

The Isanoura Park course is 2.1 km long with a cumulative altitude gain of 73 m. It passes through lush natural forests surrounding a dam lake upstream of the Isanoura River and features seasonal changes in scenery (Fig 2).

### Walking protocol

The Kurort Health Walking sessions were conducted under the guidance of two certified health and exercise trainers. The same walking protocol was applied to both courses. Each session lasted approximately 120 minutes, including warm-up exercises, the walking session, and cool-down exercises after returning to the starting point. Participants monitored their pulse rate (PR) by manual palpation at designated points along the course to maintain the target exercise intensity. The target PR was set at “160 – age” (corresponding approximately to 55%–60% of the age-predicted maximal heart rate), with the target PR reduced by 10%–20% for those receiving antihypertensive medication. Manual pulse monitoring was used solely for real-time intensity adjustments and was not included in the outcome analysis.

### Measurements

#### Blood pressure and pulse rate.

SBP, DBP, and PR were measured before (baseline) and after the walking session. Measurements were performed using a wrist-type blood pressure monitor (HEM-6230; Omron Corporation, Kyoto, Japan). To ensure accuracy, all measurements were recorded with the monitor positioned at heart level. Baseline measurements were taken after participants had been seated for 10 min following registration. Post-walking measurements were conducted with participants in a seated position after completing the cool-down stretching exercises and an additional 10-minute seated rest.

#### Autonomic nervous activity.

Autonomic indices, specifically the low frequency/high frequency (LF/HF) ratio and HF power, were obtained at four measurement points: at the start of the course, on the observation deck, by the waterside, and at the finish line (Figs 1 and 2). Measurements were performed with participants in a standing position. During each measurement, participants were instructed to position their face within a circular guide displayed on an iPad screen and to maintain their gaze on the screen while minimizing body and facial movements. Each measurement lasted approximately 30 seconds. A non-contact vital sensing application (Sensing Co., Ltd., Tokyo, Japan) was used to calculate autonomic indices from facial RGB videos. This system extracts pulse wave signals from subtle changes in facial blood flow luminance based on the principle of image-based Pulse Transit Time (iPTT). Previous studies have demonstrated the feasibility of using RGB cameras for pulse wave measurement and blood pressure estimation [19]. Validation studies conducted by the Tsumura Laboratory at Chiba University reported high agreement with contact- based electrocardiographic measurements, demonstrating >99% agreement for pulse rate and >90% agreement for the LF/HF ratio under controlled measurement conditions.

#### Psychological mood and emotion.

Psychological states were assessed before and after completion of the Kurort Health Walking Program using two questionnaires: the Profile of Mood States 2nd Edition (POMS2) Japanese version (adult short form) [20] and the Mood Check List-Short form.2 (MCL-S.2) [21].

The POMS2 evaluates mood states across seven subscales: anger-hostility (AH), confusion-bewilderment (CB), depression-dejection (DD), fatigue-inertia (FI), tension-anxiety (TA), vigor-activity (VA), and friendliness (F), as well as the Total Mood Disturbance (TMD) score. Raw scores were converted to T-scores for standardized interpretation. T-score between 40–59 was considered within the average, and higher T-scores indicated greater intensity of the respective mood states.

The MCL-S.2 consists of 12 items across three subscales: Pleasure, Relaxation, and “Anxiety with four items each subscales. Responses were recorded using a 7-point Likert scale ranging from “not at all” to “very much.” Each item was scored on a scale ranging from −3 to +3. For the Pleasure and Relaxation subscale, positive scores indicate more positive emotional states, whereas l for the Anxiety subscale, negative scores indicate more positive emotional states.

### Statistical analysis

Descriptive statistics were calculated as mean ± standard deviation (SD) or median and interquartile range (IQR) for each variable. The normality of the data distribution was assessed using the Kolmogorov–Smirnov test. To evaluate differences between measurements obtained before and after the walking session, the paired Student’s t-test was used for normally distributed data, whereas the Wilcoxon signed-rank or Friedman test was applied for non-normally distributed data. To evaluate the robustness of the observed blood pressure changes, a sensitivity analysis was conducted among participants who were not taking antihypertensive medication. To examine the appropriateness of pooling data from the two walking courses, baseline values and pre- to post-walking changes were compared between the two groups. Baseline values and changes in SBP, DBP, and PR were compared using the independent -samples Student’s t-test, whereas those for the LF/HF ratio and HF power were compared using the Mann–Whitney U test. Effect sizes and 95% confidence intervals were calculated for the primary outcomes. Cases with missing data were excluded only from the relevant analyses. All statistical analyses were performed using SPSS Statistics version 27.0 (IBM Corp., Armonk, NY, USA). The significance level was set at *p* < 0.05.

### Ethical compliance

This study was approved by the Ethics Committee of the Graduate School of Biomedical Sciences, Nagasaki University (Approval Nos. 24020806 and 24020806-2). All procedures were performed in accordance with the ethical standards of the Institutional Research Committee and the 1964 Declaration of Helsinki and its later amendments. Informed consent was obtained from all participants.

This study was reported in accordance with the Strengthening the Reporting of Observational Studies in Epidemiology (STROBE) guidelines, and the STROBE checklist is provided as Supplementary Material.

## Results

**Fig 3:**
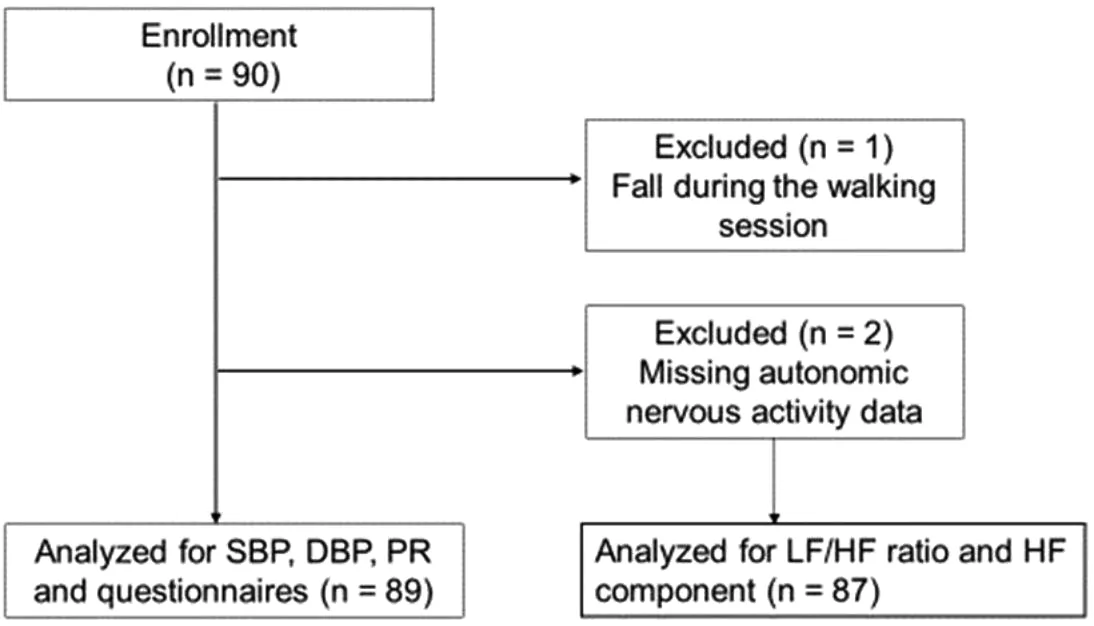
Participant flow diagram. Note: SBP, systolic blood pressure; DBP, diastolic blood pressure; PR, pulse rate; LF/HF, low-frequency/high-frequency ratio; HF, high-frequency component.

A total of 90 individuals participated in this study. One participant who fell during the Kurort Health Walking program and could not complete the session was excluded, leaving 89 participants for the analysis of blood pressure, pulse rate, and psychological measures. Of these, two individuals with missing autonomic nervous system data were excluded, resulting in 87 participants for the analysis of LF/HF ratio and HF indices (Fig 3).

Of the 89 participants analyzed, 36 were men (40.4%) and 53 were women (59.6%). The participants’ ages ranged from 18 to 83 years (mean age, 50.8 ± 16.5 years). Sixteen participants (18.0%) reported currently using antihypertensive medications. In terms of the study site, 61 participants (68.5%) completed the program at the Shihondo Park course and 28 (31.5%) at the Isanoura Park course.

Baseline characteristics did not differ significantly between participants in the Shihondo Park and Isanoura Park courses (S1 Table). In addition, no significant differences were observed between the two courses in changes in SBP, DBP, LF/HF ratio, or HF. Although changes in pulse rate differed significantly between courses (p = 0.004), no significant differences were observed in the principal physiological and autonomic outcomes. Therefore, data from the two courses were pooled for the subsequent analyses.

### SBP, DBP, and PR

SBP decreased significantly from 127.2 ± 15.9 mmHg to 120.6 ± 15.5 mmHg (mean difference: −6.6 mmHg, 95% CI: −9.2 to −4.0, *p* < 0.001, Cohen’s *d* = 0.54). DBP also decreased significantly from 81.2 ± 12.5 mmHg to 76.3 ± 13.8 mmHg (mean difference: −4.9 mmHg, 95% CI: −7.2 to −2.7, *p* < 0.001, Cohen’s *d* = 0.46). PR did not change significantly after the walking session (mean difference: 1.9 beats/min, 95% CI: −0.2 to 3.9, *p* = 0.076) (Table 1).

**Table 1:** Blood pressure and pulse rate before and after Kurort Health Walking program (n = 89).

Variable	Pre Mean ± SD	Post Mean ± SD	Mean Difference^†^	95% CI	p value*	Cohen’s d
SBP (mmHg)	127.2 ± 15.9	120.6 ± 15.5	−6.6	−9.2 to −4.0	<0.001	0.54
DBP (mmHg)	81.2 ± 12.5	76.3 ± 13.8	−4.9	−7.2 to −2.7	<0.001	0.46
PR (beats/min)	76.3 ± 10.2	78.2 ± 11.1	1.9	−0.2 to 3.9	0.076	0.19

†Mean difference was calculated as post-walking minus pre-walking values.

*Paired Student’s t-test, p < 0.05.

Note: SD, standard deviation; CI, confidence interval; SBP, systolic blood pressure; DBP, diastolic blood pressure; PR, pulse rate.

Sensitivity analysis among participants not taking antihypertensive medication demonstrated significant reductions in both SBP and DBP after the walking session, whereas no significant change was observed in PR (Table 2). These findings were consistent with those observed in the overall study population.

**Table 2:** Blood pressure and pulse rate before and after the Kurort Health Walking program among participants not taking antihypertensive medication (n = 73).

Variable	Pre Mean ± SD	Post Mean ± SD	Mean Difference^†^	95% CI	p value*	Cohen’s d
SBP (mmHg)	124.9 ± 16.2	119.5 ± 15.4	−5.4	−8.2 to −2.6	<0.001	0.46
DBP (mmHg)	79.8 ± 12.3	75.8 ± 14.1	−4.0	−6.4 to −1.5	0.002	0.38
PR (beats/min)	77.1 ± 9.1	79.2 ± 11.1	2.1	−0.1 to 4.2	0.056	0.23

†Mean difference was calculated as post-walking minus pre-walking values.

*Paired Student’s t-test. p < 0.05.

Note: SD, standard deviation; CI, confidence interval; SBP, systolic blood pressure; DBP, diastolic blood pressure; PR, pulse rate.

### Autonomic nervous system activity (LF/HF ratio and HF power)

**Fig 4:**
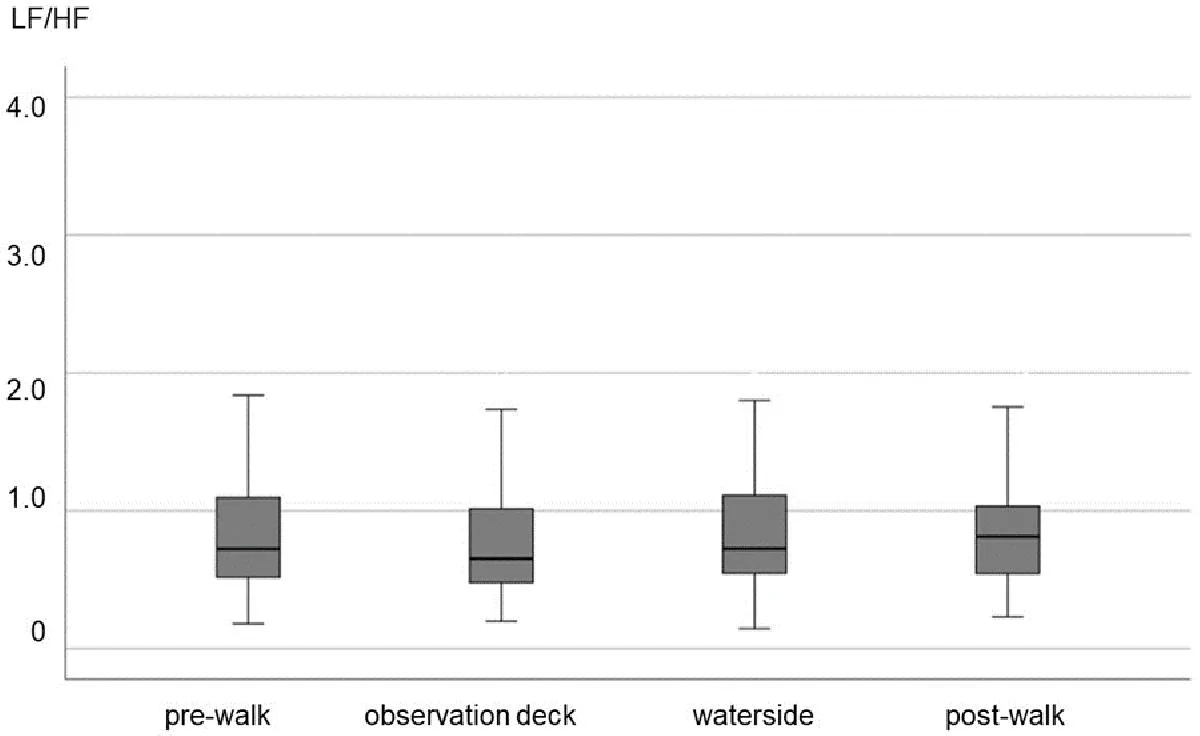
Changes in LF/HF ratio across four measurement points during Kurort Health Walking. Note: LF/HF, low-frequency/high-frequency ratio.

The LF/HF ratio across the four measurement points is shown in Fig 4. The Friedman test revealed no significant differences in the LF/HF ratio or HF power across the four measurement points (Table 3).

**Table 3:** LF/HF ratio and HF power at four measurement points during Kurort Health Walking (n = 87*).

		Median	IQR	p-value**
LF/HF	1. pre-walk	0.725	0.518–1.112	0.258
	2. observation deck	0.655	0.478–1.024	
	3. waterside	0.726	0.533–1.144	
	4. post-walk	0.814	0.536–1.043	
HF	1. pre-walk	27.77	18.60–43.32	0.527
	2. observation deck	33.41	19.17–46.64	
	3. waterside	29.18	29.18–46.59	
	4. post-walk	32.22	18.44–47.38	

*Excluded (n = 2): missing post-walk data. **Friedman test p < 0.05.

Note: IQR, interquartile range; LF/HF, low-frequency/high-frequency ratio; HF, high-frequency.

### Psychological mood and emotion

Wilcoxon signed-rank tests for POMS2 T-scores showed significant post-walking decreases in the following negative mood subscales: AH, CB, DD, FI, TA, and TMD. Conversely, F and VA scores increased significantly (Table 4). Cronbach’s alpha coefficients for the POMS2 subscales were 0.90 pre-walk and 0.84 post-walk.

**Table 4:** POMS2 subscale T-scores before and after Kurort Health Walking (n = 89).

		Median	IQR	p-value*
Anger-Hostility: AH	Pre	41.0	38.0–49.5	<0.001
	Post	38.0	37.0–39.0	
Confusion-Bewilderment: CB	Pre	46.0	40.0–53.0	<0.001
	Post	40.0	38.0–43.0	
Depression-Dejection: DD	Pre	43.0	41.0–51.0	<0.001
	Post	41.0	40.0–43.5	
Fatigue-Inertia: FI	Pre	43.0	37.0–47.0	<0.001
	Post	37.0	35.0–41.0	
Tension-Anxiety: TA	Pre	43.0	38.0–50.0	<0.001
	Post	36.0	34.0–39.0	
Vigor-Activity: VA	Pre	57.0	49.0–61.0	<0.001
	Post	61.0	55.0–69.0	
Friendliness: F	Pre	54.0	48.0–62.0	<0.001
	Post	59.0	48.0–67.0	
Total Mood Disturbance: TMD	Pre	43.0	37.0–48.0	<0.001
	Post	35.0	33.0–39.0	

*Wilcoxon signed-rank test p < 0.05.

Note: POMS2, Profile of Mood States 2nd Edition; IQR, interquartile range.

For MCL-S.2, Wilcoxon signed-rank tests revealed that the scores for Pleasure and Relaxation increased significantly, while Anxiety scores decreased significantly after the walk (Table 5). Cronbach’s alpha for the subscales ranged from 0.79 to 0.90 pre-walk and 0.80 to 0.92, post-walk.

**Table 5:** MCL-S.2 scores before and after Kurort Health Walking (n = 89).

		Median	IQR	p*-*value*
Pleasure	Pre	4.0	1.0–7.0	<0.001
	Post	8.0	4.0–10.0	
Relaxation	Pre	6.0	3.0–8.0	<0.001
	Post	8.0	5.0–11.0	
Anxiety	Pre	–8.0	–12.0 to –3.0	<0.001
	Post	–12.0	–12.0 to –8.0	

*Wilcoxon signed-rank test p < 0.05.

Note: MCL-S.2, Mood Check List-Short form.2; IQR, interquartile range.

## Discussion

The primary findings of this study are as follows: (1) participation in the community-based Kurort Health Walking program was associated with significant decreases in systolic and diastolic blood pressure, and (2) negative mood states decreased while positive emotional states, including vigor, pleasure, and relaxation, improved following participation in the program.

### Changes in blood pressure, pulse rate, and autonomic nervous activity associated with Kurort Health Walking

In the present study, SBP decreased by an average of 6.6 mmHg, DBP decreased by 4.9 mmHg, and PR showed a small increase of 1.9 bpm. These results are consistent with previous studies that investigated the effects of Kurort Health Walking [14,15]. Kurort Health Walking was conducted under the supervision of two certified health and exercise trainers. The target PR was calculated using the formula “160 − age” per minute, and participants adjusted their walking speed by monitoring their PR via manual self-palpation after passing through physically demanding points on the course. This self-regulated intensity control may have attenuated excessive increases in blood pressure and heart rate. Aerobic exercise, including walking, has been shown to lower blood pressure [22]. In addition, previous studies suggest that exposure to forest environments can exert blood-pressure-lowering effects [8]. Therefore, the observed blood pressure changes may be associated with the combined influences of aerobic exercise and the forest environment, although their independent contributions could not be distinguished in the present study.

Autonomic nervous system indices provide additional information regarding physiological responses during the walking session. In the present study, neither the LF/HF ratio nor HF power showed significant differences across the four measurement points. Previous studies have reported lower LF/HF values under relatively relaxed physiological conditions [23]; however, the LF/HF ratio should not be interpreted as a direct indicator of relaxation or sympathovagal balance. Recent studies have highlighted limitations in such interpretations because the LF component reflects contributions from both sympathetic and parasympathetic activity, and the interaction between the two branches of the autonomic nervous system is complex and non-linear [24]. Therefore, although the LF/HF values observed in the present study can be considered in the context of previous findings [23], they should not be interpreted as evidence of a specific state of autonomic balance or relaxation.

Interestingly, these findings contrast with conventional exercise physiology. Previous studies have reported that exercise at 60% intensity typically leads to increased sympathetic activity and decreased parasympathetic activity [25]. However, the results of the present health walking protocol (55%–60% intensity) did not demonstrate these earlier findings. This discrepancy may be explained by the environmental context. Exposure to forest environments significantly increases parasympathetic activity and decreases sympathetic activity [8]. Because all of the present measurement points were located within forest settings, the natural environment may have influenced the physiological responses observed during the walking session. However, the independent effects of the forest environment and exercise intensity could not be distinguished in the present study. Previous studies comparing 15-minute walks in forest and urban environments have demonstrated that forest walking significantly enhances parasympathetic activity compared with urban walking [26,27]. To further clarify the environmental effects observed in this study, future research should incorporate urban settings as controls to evaluate the comparative impact of different environments on autonomic nervous function before and after walking.

Although no significant differences were observed in HF across the four measurement points, the median values during and after the walking session were slightly higher than the baseline value. These findings may suggest that parasympathetic activity was relatively preserved during the walking session. Previous studies have suggested that positive social interactions may help preserve parasympathetic activity under conditions of low stress and high social safety [28]. Because many participants undertook Kurort Health Walking with family members, friends, partners, and colleagues, social interactions during the walking session may have contributed to maintaining parasympathetic activity. However, this interpretation remains speculative and warrants further investigation. In addition, because post-walking blood pressure measurements were obtained following stretching exercises and a 10-minute seated rest period, the observed reductions in blood pressure may partly reflect normal post-exercise hypotension.

### Psychological changes in mood states associated with Kurort Health Walking

Significant improvements were observed after Kurort Health Walking in negative mood states, including AH, CB, DD, FI, and TA, as well as in TMD scores. In addition, significant increases were observed in positive emotional states, including F, VA, pleasure, and relaxation. These findings indicate that participation in Kurort Health Walking was associated with improvements in psychological measures, consistent with previous research [17]. Notably, this study confirmed significant differences in “Friendliness”, a scale added to the POMS2, and in the standardized TMD score. Furthermore, all subscales of the MCL-S.2 showed improvement, consistent with previous studies [14–16]. Recent reviews have indicated that increases in positive affect are associated with indices reflecting parasympathetic activity [29], suggesting that mood enhancement derived from physical activity may involve complex interaction between psychological and physiological processes. Additionally, natural environments encompassing green and blue spaces (waterfronts) have been shown to be associated with mental well-being, contributing to improved mental health through psychological restoration and social interaction [30]. The two courses used for Kurort Health Walking in this study included coastal and lakeside paths, and these environmental features may have contributed to the observed improvements in psychological measures. Moreover, a review of the health impacts of nature-based outdoor activities found that reductions in anxiety and improvements in positive affect were most pronounced when activities were performed in groups [31]. In the present study, Kurort Health Walking was conducted in groups of approximately four to ten participants. Future research should further investigate the specific impact of social interaction on psychological indices in the context of Kurort Health Walking.

### Limitations and future perspectives

This study has several limitations that should be considered.

First, as this was a single-group observational pre–post study without a comparison group, it is difficult to establish a direct causal relationship between participation in the walking program and the observed physiological and psychological changes. The potential influences of physical activity, exposure to the forest environment, social interaction, expectancy effects, regression to the mean, and post-exercise recovery could not be distinguished in the present study. Future studies using controlled study designs or randomized controlled trials are needed to more rigorously evaluate the physiological and psychological changes associated with participation in Kurort Health Walking.

Second, the study population was limited to individuals who voluntarily participated in a local government health program, which may have introduced selection bias toward individuals with higher health consciousness.

Third, because the measurements were obtained only before and after a single session, the long-term effects of sustained participation in the Kurort Health Walking program remain unclear.

Fourth, autonomic nervous system activity was evaluated using the LF/HF ratio and HF power obtained with a non-contact RGB camera-based system rather than conventional ECG-derived HRV. Although previous validation studies have reported high agreement with contact-based measurements under controlled conditions, measurements obtained in field settings may be susceptible to body and facial movements, environmental conditions, and other motion-related artifacts. In addition, the approximately 30-second measurement duration used in this study was shorter than that commonly used for conventional ECG-based HRV assessment. Therefore, the autonomic indices obtained using this system should not be considered equivalent to those derived from conventional ECG-based HRV assessment. Furthermore, recent studies have highlighted limitations in interpreting the LF/HF ratio as a direct measure of sympathovagal balance. Heart rate variability indices are influenced by multiple factors, including respiration, posture, physical activity, and measurement conditions. Therefore, the LF/HF ratio alone may not provide a comprehensive assessment of autonomic nervous system function. Accordingly, the present findings should be interpreted as indicating the absence of marked sympathetic predominance during the walking session rather than providing definitive evidence of autonomic balance or relaxation.

Finally, because multiple physiological and psychological outcomes were evaluated in this exploratory study, the possibility of an increased Type I error rate due to multiple statistical comparisons cannot be excluded. Accordingly, the present findings should be interpreted with appropriate caution.

Despite these limitations, this study provides real-world data on the short-term physiological and psychological changes associated with participation in a community-based Kurort Health Walking program conducted in a forest environment. Future longitudinal studies involving larger and more diverse populations are warranted to validate these findings and contribute to the development of more effective and individualized health promotion programs.

## Conclusions

This study found that participation in a community-based Kurort Health Walking program conducted in a forest environment was associated with favorable short-term physiological and psychological changes, including reductions in blood pressure and improvements in psychological measures. No significant changes in autonomic nervous system indices were observed. These findings provide preliminary insights regarding physiological and psychological responses associated with participation in a structured community-based walking program conducted in a natural environment. Further controlled studies are needed to determine the independent contributions of physical activity, the forest environment, and other contextual factors.

## Supporting information

S1 Table Baseline characteristics and physiological variables according to walking course. This table presents baseline characteristics and physiological variables of participants according to the Shihondo Park and Isanoura Park courses.
(DOCX)

S2 Table Changes in physiological and autonomic outcomes according to walking course. This table presents changes in physiological and autonomic outcomes according to walking course, comparing the Shihondo Park and Isanoura Park courses.
(DOCX)

S1 Data Minimal dataset underlying the findings of this study. This file contains the minimal dataset underlying the physiological and psychological outcomes reported in this study, including blood pressure, pulse rate, autonomic nervous system indices, and psychological measures.
(XLSX)
