# Supplementary material for: Physiological and psychological outcomes of a community-based Kurort Health Walking program: A before–after study
Source: PLoS One. 2026 Sep 3;21(9):e0357733. doi: 10.1371/journal.pone.0357733 (PMC13541170; doi:10.1371/journal.pone.0357733)
Supplement: Supplementary file 1 [file pone.0357733.s004.pdf]

I would like to thank the authors for the opportunity to review this manuscript. The topic is relevant and the study explores an interesting community-based health promotion program. However, several methodological and interpretative issues should be addressed before the manuscript can be considered for publication.

Major Comments

1. Lack of a control group limits causal inference

The study uses a pre–post observational design without a control group. Consequently, it is not possible to distinguish the effects of:

- physical activity itself,
- exposure to the forest environment,
- social interaction among participants,
- expectancy/placebo effects,
- regression to the mean,
- or post-exercise recovery.

The authors acknowledge this limitation in the conclusion (Lines 30–33), but throughout the Discussion they continue to attribute improvements to the forest environment and the Kurort Health Walking program.

The conclusions should therefore be more cautious and limited to reporting associations rather than implying causal effects.

2. Overinterpretation of LF/HF ratio

The manuscript repeatedly interprets LF/HF values below 2.0 as evidence of a "deeply relaxed autonomic state" (Lines 213–215).

This interpretation is problematic because:

- LF/HF is no longer universally accepted as a direct measure of sympathovagal balance.
- There is no widely accepted physiological threshold defining LF/HF < 2 as "deep relaxation."
- Contemporary HRV literature recommends caution when assigning physiological meaning to LF/HF values.

Although the authors later acknowledge some limitations of LF/HF interpretation (Lines 265–271), they continue to use this index as evidence of autonomic relaxation throughout the manuscript.

The interpretation of LF/HF should be substantially revised.

3. Validity of autonomic nervous system measurements

Autonomic activity was assessed using a non-contact RGB camera application rather than conventional ECG-derived HRV (Lines 132–139).

Additional discussion is needed regarding:

- measurement validity,
- reliability in field conditions,
- susceptibility to motion artifacts,
- limitations compared with ECG-based HRV assessment.

The current description emphasizes validation but does not adequately discuss the limitations of this methodology.

4. Blood pressure reductions may reflect post-exercise hypotension

Blood pressure measurements were obtained after:

- completion of walking,
- stretching,
- and an additional 10-minute seated recovery period (Lines 125–127).

Therefore, the observed reductions in SBP and DBP may reflect normal post-exercise hypotension rather than a specific effect of the Kurort Health Walking program or forest environment.

This limitation should be discussed more explicitly.

5. Antihypertensive medication as a confounding factor

The manuscript reports that 16 participants (18.0%) were taking antihypertensive medication (Lines 190–191).

However:

- no subgroup analyses were performed,
- no sensitivity analyses were reported,
- no statistical adjustment was conducted.

Because blood pressure is a primary outcome, the potential influence of antihypertensive medication should be addressed.

6. Multiple comparisons without correction

The study evaluates numerous outcomes:

- SBP,
- DBP,
- pulse rate,
- LF/HF,
- HF,
- eight POMS2 outcomes,
- three MCL-S.2 outcomes.

No adjustment for multiple testing is reported (Lines 156–164).

The authors should justify this approach or discuss the increased risk of Type I error.

7. Pooling data from two different courses

The manuscript states that participants completed the intervention in two different locations (Shihondo Park and Isanoura Park) and that baseline characteristics and primary outcomes were compared between groups before pooling the data (Lines 191–196).

However:

- the statistical results supporting this decision are not presented;
- the statistical tests used are not reported;
- exact p-values are not provided;
- effect sizes are not reported.

The authors state that:

“No significant differences were observed…” (Lines 194–196)

However, absence of statistical significance should not automatically be interpreted as evidence of equivalence.

Because the two courses appear to differ in environmental characteristics (coastal landscape versus forest-lake environment; Lines 91–103), additional justification for pooling the data is warranted.

The authors should provide the actual comparison results, preferably in a supplementary table.

8. Contradictory interpretation of autonomic outcomes

The manuscript concludes that participants maintained a relaxed autonomic state throughout the intervention because LF/HF remained below 2.0 (Lines 208–215). However, Table 2 shows that neither LF/HF nor HF changed significantly across measurement points (Lines 218–222).

Therefore, the results may simply indicate a lack of measurable autonomic change rather than evidence that the intervention induced autonomic relaxation.

This distinction should be clarified.

9. Lack of effect size reporting

The manuscript reports statistical significance but does not report effect sizes or confidence intervals for primary outcomes (Lines 198–205).
Reporting effect sizes would substantially improve interpretation of the practical and clinical significance of the findings.

10. Pulse rate versus heart rate terminology

The manuscript uses pulse rate (PR) as an outcome measure (Lines 121–123) while discussing autonomic responses based on HRV-derived indices (Lines 130–139).

The methods section would benefit from a clearer explanation distinguishing:

- pulse rate measured by the blood pressure monitor,
- pulse rate used during exercise intensity monitoring,
- autonomic indices derived from facial video recordings.

Minor Comments

11. STROBE reporting

Although the manuscript states adherence to STROBE guidelines (Lines 173–175), several methodological details remain insufficiently reported, particularly regarding participant selection, missing data handling, and justification for pooling the two course groups.

12. Language of conclusions

The conclusion currently states that participation in the program "may promote physiological and psychological well-being" (Lines 30–32).

Given the observational pre–post design without a control group, a more appropriate conclusion would be that participation was **associated with** improvements in physiological and psychological measures.

Overall Recommendation

The manuscript addresses an important public health topic and presents interesting findings. However, substantial revisions are needed regarding interpretation of autonomic outcomes, methodological transparency, statistical reporting, and causal inference.
