# Supplementary material for: Physiological and psychological outcomes of a community-based Kurort Health Walking program: A before–after study
Source: PLoS One. 2026 Sep 3;21(9):e0357733. doi: 10.1371/journal.pone.0357733 (PMC13541170; doi:10.1371/journal.pone.0357733)
Supplement: Supplementary file 2 [file pone.0357733.s001.docx]

Supplementary Table S1

Baseline characteristics and physiological variables according to walking course

| Variable | Shihondo Park (n = 61) | Isanoura Park (n = 28) | p-value |
| --- | --- | --- | --- |
| Age, years | 52.4±16.6 | 47.3±15.9 | 0.168 |
| Female, n (%) | 38（62.3） | 15（53.6） | 0.436 |
| SBP, mmHg | 128.9±15.1 | 123.4±17.2 | 0.132 |
| DBP, mmHg | 81.8±12.6 | 79.4±12.2 | 0.518 |
| PR | 74.9±10.5 | 79.4±8.9 | 0.053 |
| LF/HF (Start) | 0.72(0.52-1.13) | 0.65 (0.44-1.05) | 0.230 |
| HF (Start) | 27.39(15.90-39.86) | 32.67(20.67-54.67) | 0.424 |

Note. Data are presented as mean ± SD or median (interquartile range [IQR]). p-values were calculated using the chi-square test for sex, the independent-samples t test for systolic blood pressure, diastolic blood pressure, and pulse rate variables, and the Mann–Whitney U test for LF/HF ratio and HF variables. A two-sided p value < 0.05 was considered statistically significant.
