# Supplementary material for: Physiological and psychological outcomes of a community-based Kurort Health Walking program: A before–after study
Source: PLoS One. 2026 Sep 3;21(9):e0357733. doi: 10.1371/journal.pone.0357733 (PMC13541170; doi:10.1371/journal.pone.0357733)
Supplement: Supplementary file 3 [file pone.0357733.s002.docx]

Supplementary Table S2

Changes in physiological and autonomic outcomes according to walking course

| Variable | Shihondo Park | Isanoura Park | p-value |
| --- | --- | --- | --- |
| ΔSBP (mmHg) | -6.82±12.6 | -6.11±11.8 | 0.801 |
| ΔDBP (mmHg) | -5.39±11.1 | -3.93±9.7 | 0.549 |
| ΔPR | 3.80±9.9 | -2.43±7.6 | 0.004 |
| LF/HF (Start) | 0.72(0.52-1.13) | 0.65 (0.44-1.05) | 0.230 |
| LF/HF (Observation deck) | 0.66(0.45-1.08) | 0.60(0.54-0.96) | 0.711 |
| LF/HF (Waterside) | 0.72(0.51-1.17) | 0.75(0.53-1.10) | 0.665 |
| LF/HF (Finish) | 0.84(0.53-1.00) | 0.74(0.54-1.07) | 0.892 |
| ΔLF/HF | 0.07(-0.38-0.38) | 0.11(-0.26-0.58) | 0.424 |
| HF (Start) | 27.39(15.90-39.86) | 32.67(20.67-54.67) | 0.069 |
| HF (Observation deck) | 34.51(19.82-53.39) | 27.45(23.09-44.69) | 0.442 |
| HF (Waterside) | 29.12(20.03-48.71) | 30.50(16.51-37.54) | 0.179 |
| HF (Finish) | 31.72(16.62-43.65) | 34.48(21.20-50.24) | 0.234 |
| ΔHF | 3.15(-10.40-21.79) | 2.31(-24.77-16.63) | 0.637 |

Note. Data are presented as mean ± SD or median (interquartile range [IQR]). Changes (Δ) were calculated as post-walking values minus pre-walking values (post−Pre); therefore, negative values indicate decreases after walking. To assess the appropriateness of pooling data from the two courses, changes in systolic blood pressure, diastolic blood pressure, and pulse rate were compared between the courses using independent-samples t tests, whereas changes in LF/HF ratio and HF power were compared using the Mann–Whitney U test. A two-sided p value < 0.05 was considered statistically significant.
