# Supplementary material for: Physiological and psychological outcomes of a community-based Kurort Health Walking program: A before–after study
Source: PLoS One. 2026 Sep 3;21(9):e0357733. doi: 10.1371/journal.pone.0357733 (PMC13541170; doi:10.1371/journal.pone.0357733)
Supplement: Supplementary file 4 [file pone.0357733.s006.docx]

Dear Editor and Reviewers,

We sincerely thank you for your careful review of our manuscript entitled “Physiological and psychological outcomes of a community-based Kurort Health Walking program: a before–after study” (Manuscript ID: PONE-D-26-23903). We greatly appreciate the constructive and insightful comments provided by the Editor and Reviewers, which have helped us improve the clarity and scientific rigor of our manuscript.

In response to the comments, we have thoroughly revised the manuscript. The major revisions are summarized as follows:

1. We clarified the study design as a single-group observational pre–post study conducted within an existing community-based public health program and provided additional details regarding participant recruitment, inclusion and exclusion criteria, and informed consent procedures.
2. We expanded the description of the walking protocol and measurement procedures by specifying the duration of the intervention, exercise intensity, pulse rate monitoring procedures, body position during measurements, and the timing of post-walking assessments, including stretching exercises and a 10-minute seated rest period.
3. We substantially revised the interpretation of autonomic nervous system activity to avoid overinterpreting the LF/HF ratio as a direct indicator of sympathovagal balance or relaxation. We removed interpretations based on an LF/HF ratio below 2.0 as evidence of a relaxed autonomic state and now focus on the finding that neither the LF/HF ratio nor HF power showed significant differences across the measurement points. We also expanded the discussion of the limitations of LF/HF-based interpretation.
4. We added a sensitivity analysis among participants who were not taking antihypertensive medication to evaluate the robustness of the observed blood pressure changes. In addition, we conducted additional analyses comparing the two Kurort Health Walking courses. Baseline characteristics and physiological indices are presented in Supplementary Table S1, while between-course comparisons of changes in physiological and autonomic indices and autonomic indices at each measurement point are presented in Supplementary Table S2. These analyses were used to assess the appropriateness of pooling data from the two courses.
5. We added effect sizes and 95% confidence intervals for the primary outcomes and clarified the handling of missing data in accordance with the STROBE guidelines.
6. We revised the Abstract, Discussion, Limitations, and Conclusions to avoid implying causal relationships between participation in the walking program and the observed outcomes. Throughout the manuscript, causal expressions were replaced with language describing associations. We also clarified that the observed changes could not be attributed independently to physical activity, the forest environment, social interaction, expectancy effects, post-exercise recovery, or other contextual factors within the present study design.
7. We strengthened the discussion of study limitations by addressing the lack of a comparison group, potential selection bias among voluntary participants, the possibility of post-exercise hypotension, limitations in the interpretation and measurement of autonomic nervous system activity, and the increased risk of Type I error due to multiple statistical comparisons. Future directions for controlled and longitudinal studies are also discussed.
8. We revised the manuscript to improve compliance with the STROBE reporting guidelines and have provided the completed STROBE checklist as Supplementary Material.
9. We prepared all figures included in the revised manuscript using copyright-free materials and resources distributed under licenses permitting reuse and adaptation. Appropriate attribution has been provided where required, and no additional permission for reproduction was necessary.

We believe that these revisions have substantially improved the scientific rigor, transparency, and clarity of the manuscript. We are grateful for the Editor's and Reviewers' valuable comments and hope that the revised manuscript is now suitable for publication in PLOS ONE.

Sincerely,

Saori Miura, on behalf of all authors

Major Comment

1. Lack of a control group limits causal inference

Response:

We thank the Reviewer for this important comment. We agree that, because this study used a single-group observational pre–post design without a comparison group, the observed physiological and psychological changes cannot be attributed specifically to Kurort Health Walking or to the forest environment.

In response to this comment, we revised the manuscript to address the potential contributions identified by the Reviewer. Specifically, the observed changes may have been influenced by physical activity itself as well as exposure to the forest environment, and their independent contributions could not be distinguished in the present study (Lines 290-292). We also expanded the Discussion to acknowledge that social interaction during group walking may have contributed to the observed psychological and autonomic responses (Lines 321-325). In addition, because post-walking blood pressure was measured following stretching exercises and a 10-minute seated rest period, we now acknowledge that the observed blood pressure reductions may partly reflect normal post-exercise hypotension and recovery (Lines 326-328).

We also recognize that, in the absence of a comparison group, expectancy or placebo effects and regression to the mean cannot be excluded as alternative explanations for the observed changes. These factors cannot be separately evaluated using the present study design and therefore represent additional reasons why causal inference should be avoided.

Accordingly, we revised the Abstract, Discussion, Limitations, and Conclusions to avoid causal language and to describe the findings in terms of associations. For example, the Conclusions now state that participation in the community-based Kurort Health Walking program "was associated with" favorable short-term physiological and psychological changes (Lines 393-395). The Limitations section also explicitly acknowledges that the lack of a comparison group prevents establishment of a direct causal relationship and emphasizes the need for future controlled studies to distinguish the contributions of physical activity, environmental exposure, social interaction, expectancy effects, and post-exercise recovery (Lines 357-361).

2. Overinterpretation of LF/HF ratio

Response:

We thank the Reviewer for this important comment regarding the interpretation of autonomic nervous system indices. In response, we substantially revised the manuscript to avoid overinterpreting the LF/HF ratio as a direct indicator of sympathovagal balance or relaxation. Specifically, we removed statements interpreting an LF/HF ratio below 2.0 as evidence of a deeply relaxed or sustained autonomic relaxation state and revised the Abstract, Results, Discussion, figure legend, and Conclusions accordingly.

The revised manuscript now focuses on the observed finding that neither the LF/HF ratio nor HF power showed significant differences across the four measurement points, without assigning a specific autonomic state to the LF/HF values. In the Discussion, we retained previous findings reporting lower LF/HF values under relatively relaxed physiological conditions as contextual information, while explicitly clarifying that such observations do not establish the LF/HF ratio as a direct indicator of relaxation or sympathovagal balance.

We also expanded the Discussion and Limitations sections to acknowledge current concerns regarding the physiological interpretation of the LF/HF ratio, including the contribution of both sympathetic and parasympathetic activity to the LF component and the complex, non-linear interaction between the two branches of the autonomic nervous system. Accordingly, the revised manuscript interprets the autonomic findings cautiously and does not use the LF/HF ratio as evidence of a specific state of autonomic balance or relaxation.

3. Validity of autonomic nervous system measurements

Response:

We thank the Reviewer for this important comment. We agree that the limitations of the non-contact RGB camera-based assessment of autonomic nervous activity should be more clearly addressed.

We have expanded the description of the autonomic nervous system measurements in the Methods section by adding details regarding the standing measurement position, measurement duration (approximately 30 seconds), facial positioning procedures using an iPad display, and the principle underlying the image-based Pulse Transit Time (iPTT) method (Lines 141-148). We also clarified the previously reported validation results of this non-contact measurement system (Lines 148-149).

In addition, we have revised the Discussion and Limitations sections to acknowledge methodological limitations of the non-contact measurements. Specifically, we now note that measurements obtained under field conditions may be affected by body and facial movements, environmental conditions, and other sources of motion artifact; that the approximately 30-second measurement duration is shorter than that typically used for conventional ECG-based heart rate variability assessment; and that the non-contact system should not be regarded as equivalent to standard ECG-derived HRV measurements (Lines 367-375). Accordingly, the LF/HF ratio and HF power obtained in this study were interpreted cautiously as exploratory indices of autonomic nervous activity rather than definitive measures of sympathovagal balance.

4. Blood pressure reductions may reflect post-exercise hypotension

Response:

We thank the Reviewer for this important suggestion. We have clarified the timing of post-walking measurements in the Methods section, including cool-down stretching exercises and a subsequent 10-minute seated rest period (Lines 134-136). In addition, we have revised the Discussion section to acknowledge that the observed reductions in blood pressure may partly reflect normal post-exercise hypotension following physical activity (Lines 326-328). We believe this revision provides a more balanced interpretation of the physiological findings.

5. Antihypertensive medication as a confounding factor

Response:

To address this concern, we conducted a sensitivity analysis among participants who were not taking antihypertensive medication. The results demonstrated significant reductions in both systolic and diastolic blood pressure after the walking session, which were consistent with the findings observed in the overall study population. These additional analyses have been incorporated into the Results (Lines 231-240) and Statistical Analysis sections (Lines 176-177).

6. Multiple comparisons without correction

Response:

We thank the Reviewer for this important comment. We acknowledge that multiple physiological and psychological outcomes were evaluated in the present study and that no adjustment for multiple comparisons was applied. We consulted with a statistical expert regarding the appropriate approach to multiple comparisons in the context of the present study. Given the exploratory nature of this study, we retained the original statistical approach without applying a formal adjustment for multiple comparisons. However, we agree that conducting multiple statistical tests may increase the risk of Type I error. Accordingly, we have added this issue to the Limitations section and clarified that the findings should be interpreted with appropriate caution in light of the potential for an increased Type I error rate due to multiple comparisons (Lines 382–385).

7. Pooling data from two different courses

Response:

We appreciate the Reviewer's suggestion. To justify pooling data from the two Kurort Health Walking courses, we conducted additional analyses comparing baseline characteristics and physiological variables between the two groups. The results are presented in Supplementary Table S1 and demonstrated no significant differences in age, sex, or baseline physiological and autonomic indices between the two courses.

Furthermore, we compared pre- to post-walking changes in physiological and autonomic outcomes between the two courses. The results are presented in Supplementary Table S2. Although changes in pulse rate differed significantly between the two courses, no significant differences were observed in changes in systolic blood pressure, diastolic blood pressure, LF/HF ratio, or HF power. In addition, LF/HF ratio and HF power at each measurement point showed no significant differences between the two courses. These findings indicate generally comparable physiological and autonomic responses across the two walking courses.

Therefore, we considered pooling the data from the two courses appropriate for the subsequent analyses.

8. Contradictory interpretation of autonomic outcomes

Response:

We substantially revised the Discussion and Limitations sections to provide a more cautious interpretation of the autonomic nervous system findings. Rather than interpreting LF/HF values as direct evidence of relaxation or autonomic balance, we now describe the findings as indicating the absence of marked sympathetic predominance at all measurement points. We also discussed the potential influence of social interaction and environmental factors while acknowledging that these interpretations remain speculative and require further investigation.

9. Lack of effect size reporting

Response:

We thank the Reviewer for this valuable suggestion. In response to this comment, we have added effect sizes and 95% confidence intervals for the primary physiological outcomes. Specifically, mean differences, 95% confidence intervals, and Cohen's d values are now reported for systolic blood pressure and diastolic blood pressure in the Results section and Table 1. In addition, 95% confidence intervals are reported for pulse rate. We have also revised the Statistical Analysis section to explicitly state that effect sizes and 95% confidence intervals were calculated for the primary outcomes (Lines 181-182). We believe that these additions improve the interpretation of both the statistical and practical significance of the observed findings.

10. Pulse rate versus heart rate terminology

Response:

We thank the Reviewer for this helpful comment. To improve clarity, we have revised the Methods section to clearly distinguish among the three physiological measurements used in this study. First, pulse rate (PR) measured using the wrist-type blood pressure monitor was used as an outcome measure and assessed before and after the walking session (Lines 131-133). Second, manual pulse palpation was used solely for real-time exercise intensity adjustment during the walking session and was not included in the outcome analysis (Lines 126-127). Third, autonomic nervous system indices (LF/HF ratio and HF power) were derived from facial RGB video recordings using a non-contact vital sensing system based on image-based Pulse Transit Time (iPTT) (Lines 144-148). These measurements were obtained independently of pulse rate measurements and were used exclusively for the assessment of autonomic nervous system activity. We believe that these revisions improve the clarity of the measurement procedures and terminology used throughout the manuscript.

Minor Comments

11. STROBE reporting

Response:

We thank the Reviewer for this helpful comment. We have revised the manuscript to improve compliance with the STROBE guidelines. Specifically, we have clarified the participant recruitment procedures, inclusion and exclusion criteria, and informed consent process in the Methods section. We have also explicitly described the handling of missing data in the Statistical Analysis section. Furthermore, to justify pooling the data from the two Kurort Health Walking courses, we conducted additional analyses comparing baseline characteristics and pre- to post-walking changes between the two groups and have provided these results in Supplementary Table S1. We believe that these revisions substantially improve the transparency and completeness of the methodological reporting.

12. Language of conclusions

Response:

We appreciate the Reviewer's important suggestion. In response to this comment, we have revised the Conclusions section to avoid implying causal relationships between participation in the program and the observed outcomes. Consistent with the observational pre–post study design, we now state that participation in the community-based Kurort Health Walking program was associated with favorable short-term physiological and psychological changes (Lines 393-395). Similar revisions have been made throughout the manuscript to ensure that the language used appropriately reflects the limitations of the study design.
